# Protection Induced by Simultaneous Subcutaneous and Endobronchial Vaccination with BCG/BCG and BCG/Adenovirus Expressing Antigen 85A against *Mycobacterium bovis* in Cattle

**DOI:** 10.1371/journal.pone.0142270

**Published:** 2015-11-06

**Authors:** Gillian S. Dean, Derek Clifford, Adam O. Whelan, Elma Z. Tchilian, Peter C. L. Beverley, Francisco J. Salguero, Zhou Xing, Hans M. Vordermeier, Bernardo Villarreal-Ramos

**Affiliations:** 1 TB Research Group, APHA Weybridge, Woodham Lane, New Haw, KT15 3NB, Surrey, United Kingdom; 2 McMaster Immunology Research Centre, Department of Pathology and Molecular Medicine, McMaster University, Hamilton, Ontario, Canada; 3 The Peter Medawar Building for Pathogen Research, University of Oxford, South Parks Road, Oxford, United Kingdom; University of Cape Town, SOUTH AFRICA

## Abstract

The incidence of bovine tuberculosis (bTB) in the GB has been increasing since the 1980s. Immunisation, alongside current control measures, has been proposed as a sustainable measure to control bTB. Immunisation with *Mycobacterium bovis* bacillus Calmette-Guerin (BCG) has been shown to protect against bTB. Furthermore, much experimental data indicates that pulmonary local immunity is important for protection against respiratory infections including *Mycobacterium tuberculosis* and that pulmonary immunisation is highly effective. Here, we evaluated protection against *M*. *bovis*, the main causative agent of bTB, conferred by BCG delivered subcutaneously, endobronchially or by the new strategy of simultaneous immunisation by both routes. We also tested simultaneous subcutaneous immunisation with BCG and endobronchial delivery of a recombinant type 5 adenovirus expressing mycobacterial antigen 85A. There was significantly reduced visible pathology in animals receiving the simultaneous BCG/BCG or BCG/Ad85 treatment compared to naïve controls. Furthermore, there were significantly fewer advanced microscopic granulomata in animals receiving BCG/Ad85A compared to naive controls. Thus, combining local and systemic immunisation limits the development of pathology, which in turn could decrease bTB transmission.

## Introduction

Infection with *Mycobacterium bovis*, the principal agent responsible for bovine tuberculosis (bTB), in the GB cattle herd is an increasing challenge to the GB farming industry. This increase is due in part to the existence of a wildlife reservoir and it has implications for both animal and human health and welfare [1]. Immunisation of cattle has been proposed for control of bTB as it is thought that the current test and slaughter policy alone will not be sufficient for eradication of the disease, in the presence of a wildlife reservoir [2].

BCG is a live attenuated strain of *M*. *bovis* that has been used as a vaccine against TB in humans since 1921 [3]. It was isolated by Calmette and Guérin in the early 1900s from a case of bTB mastitis and it has been used experimentally as a vaccine in cattle. The protection afforded by BCG has been shown to be variable in both humans and cattle [4, 5]. Furthermore, immunisation with BCG interferes with the current diagnostic assay for bTB and its use in the field is prohibited by both domestic and European legislation. Nevertheless, experimentally, BCG is the standard by which all other vaccines are judged.

A human serotype 5, replication deficient adenovirus (Ad) expressing the mycobacterial mycolyl transferase antigen Ag85A (Ad85A) [6] has been developed as a vaccine against *M*. *tuberculosis* (*Mtb*). In mice Ad85A is protective against pulmonary *Mtb* challenge when administered intranasally (i.n.) but not parenterally. Similarly after parenteral BCG priming a single i.n. but not parenteral booster immunisation with Ad85A induced improved protection against airway *Mtb* challenge compared to parenteral BCG only [7–10]

Heterologous prime-boost immunisation with parenteral BCG followed by boosting with parenteral Ad85A has also been tested in cattle and compared with BCG alone; fewer BCG primed Ad85A boosted animals presented with visible lesions after infection with *M*. *bovis* and the pathology score was reduced [11]. More recently we have shown that endonbronchial boosting of BCG vaccinated cattle with Ad85A induces immune responses similar to those induced by Ad85A [12].

Recently, it has been shown that simultaneous immunisation of mice with subcutaneous (s.c.) and i.n. BCG (SIM BCG/BCG) or BCG s.c. and recombinant antigen 85A protein (r85A) i.n., increased protection over BCG given by either route alone [13]. Protection was shown not to be due to a prime-boost effect but to inhibition of early growth of *Mtb* in the lung as a result of i.n. immunisation [14].

The experiments described here are aimed at determining whether SIM BCG/BCG or SIM BCG/Ad85A are more protective than parenteral or endobronchial delivery of BCG in cattle.

## Materials and Methods

### Cattle

The study protocol was approved by the APHA Animal Use Ethics Committee (UK Home Office PCD number 70/6905) and performed in accordance with the UK Animal (Scientific Procedures) Act 1986. Male, weaned, 5–7 months old Holstein-Friesian calves were purchased from farms known to be free of bTB. Forty nine animals were randomly divided into four groups of ten and one of nine and inoculated as described below.

### Vaccination and infection

*M*. *bovis* BCG Danish 1331 (Staten Serum Institute, Copenhagen, Denmark) was used for vaccination and was prepared as per manufacturer’s instructions (SSI, Denmark). The Ad85A construct encoding for antigen 85A has been previously described [10].

The five groups of cattle were vaccinated as follows:

Group one was inoculated with 10^6^ cfu of BCG s.c.; group two with 10^6^ cfu of BCG e.b.; group three simultaneously with 5 *X* 10^5^ cfu of BCG s.c. and 5 *X* 10^5^ cfu of BCG SSI e.b. (SIM BCG/BCG); group four simultaneously with 10^6^ cfu of BCG SSI s.c. and 2 x 10^9^ pfu Ad85A e.b. (SIM BCG/Ad85A); group five, nine animals, naïve controls, were left unimmunised.

Animals were infected twelve weeks later with 2 X 10^3^ cfu *M*. *bovis* AF2122/97 e.b. as previously described [15]

### Evaluation of immune responses

Immune responses were evaluated through the production of IFNγ using ELISA or *ex vivo* ELISpot as described below.

ELISA was used to measure secretion of IFN in whole blood as previously described [16–18]; triplicate samples were incubated overnight at 37°C with medium alone (negative control [NC]), *M*. *bovis* PPD (PPD-B) (10 μg/ml) (VLA-Weybridge, UK), Rv0288, also known as TB10.4 (10 μg/ml) (Proteix, Prague, Czech Republic), r85A protein (10 μg/ml) (Lionex, Germany) or pokeweed mitogen (PWM) Sigma-Aldrich, UK) (5 μg/ml) in an atmosphere of 5% CO_2_ and 95% humidity. After overnight incubation, blood was centrifuged at 300 x *g* for 10 min. Plasma was harvested and stored at –80°C until use. Concentration of IFN was determined using the Bovigam^™^ assay (Prionics AG, Switzerland); results were first corrected for background by subtracting unstimulated values and then expressed as mean optical density at 450 nm (O.D.) +/- standard error of the mean.

*Ex vivo* ELISpot was used to determine the frequency of IFNγ producing cells; duplicate samples of bovine PBMC at 1 x 10^6^ /ml were incubated overnight at 37°C with the same panel of antigens as was used for the whole blood assay in an atmosphere of 5% CO_2_ and 95% humidity using 96-well flat membrane-bottomed plates (Immobilon-P polyvinylidene difluoride membranes; Millipore, Ireland). Plates were coated and developed with antibodies to bovine IFNγ provided in a commercially available kit (Mabtech, Stockholm, Sweden); in accordance with the manufacturer’s instructions [19]. Results were corrected for background by subtracting unstimulated values and adjusted to express numbers of precursors per million cells.

### Gross (visible) pathology and histopathology

Twenty four weeks after BCG vaccination, cattle were euthanized and detailed *post mortem* examinations (PME) carried out as described elsewhere [20]; briefly, personnel performing postmortems were blind to the vaccination status of the animals examined. Lungs were examined externally for the occurrence of lesions, followed by slicing of the lung into 0.5- to 1-cm-thick slices that were then individually examined for lesions. In addition, lymph nodes of the head and pulmonary regions were removed, weighed and sliced into 1 to 2 mm thick sections and examined for the presence of visible lesions. Pathology scores were assigned for lungs as follows: 0 = no visible lesions; 1 = no visible lesions but lesions apparent on slicing; 2 = < 5 visible lesions of <10 mm in diameter; 3 = >6 visible lesions of <10 mm in diameter or a single distinct visible lesion of >10 mm in diameter; 4 = >1 distinct visible lesion of >10 mm in diameter; 5 = visible coalescing lesions. Individual lobe scores for each animal were added up to calculate the lung score. For lymph nodes the following criteria were applied: 0 = no necrosis or visible lesions; 1 = small focus (1 to 2 mm in diameter); 2 = several small foci or necrotic area of at least 5 by 5 mm; 3 = extensive necrosis. Individual lymph node scores were added up to calculate the lymph node score. Lung and lymph node scores were added to obtain the total pathology score for individual animal. Tissue samples were preserved in 10% phosphate buffered formalin and used for evaluation of histopathology as previously described [21]. For histopathology, sections of thoracic (caudal mediastinal, cranial mediastinal, cranial tracheobronchial, left and right bronchial) lymph nodes were used. Tissue sections were stained with haematoxilin and eosin for examination with light microscopy to assess the number, developmental stage and distribution of each granuloma (I-IV) as previously described [21, 22]. The pathologist reading the slides was blind to the distribution of the animals within the groups.

### Evaluation of bacterial load

Tissue samples collected at PME were homogenized in sterile water using a stomacher (Seward, U.K.). Viable counts were performed on serial dilutions of the macerate in PBS. Suspensions were plated on 7H10 agar plates containing sodium pyruvate (4.16 mg/ml) and 10% (vol/vol) Middlebrook oleic acid-albumin-dextrose-catalase enrichment. Plates were seeded with 500, 50 and 5 μl of macerate; therefore, the limit of detection is 2 cfu/ml. Counts were performed in preselected sections with visible lesions. However, when no lesions were found tissue samples were taken to determine possible presence of mycobacteria in the absence of lesions. Data are presented in S1 Table.

### Statistical analysis

Statistical analysis and graph drawing were carried out using GraphPad’s Instat 3 and Prism v 6.0 (GraphPad Software, CA. USA). Immune responses between groups were analysed using a Kruskal-Wallis test with Dunn’s multiple column post-test comparison. Immune response kinetics were analysed using a Friedman test with Dunn’s multiple column post-test comparison. For comparison of visible pathology and histopathology the non-parametric two tail Mann-Whitney test was used. Significance is indicated by one symbol p<0.05, on the graph; two symbols p<0.01; and three symbols p<0.001.

## Results

### Immune responses

**Fig 1:**
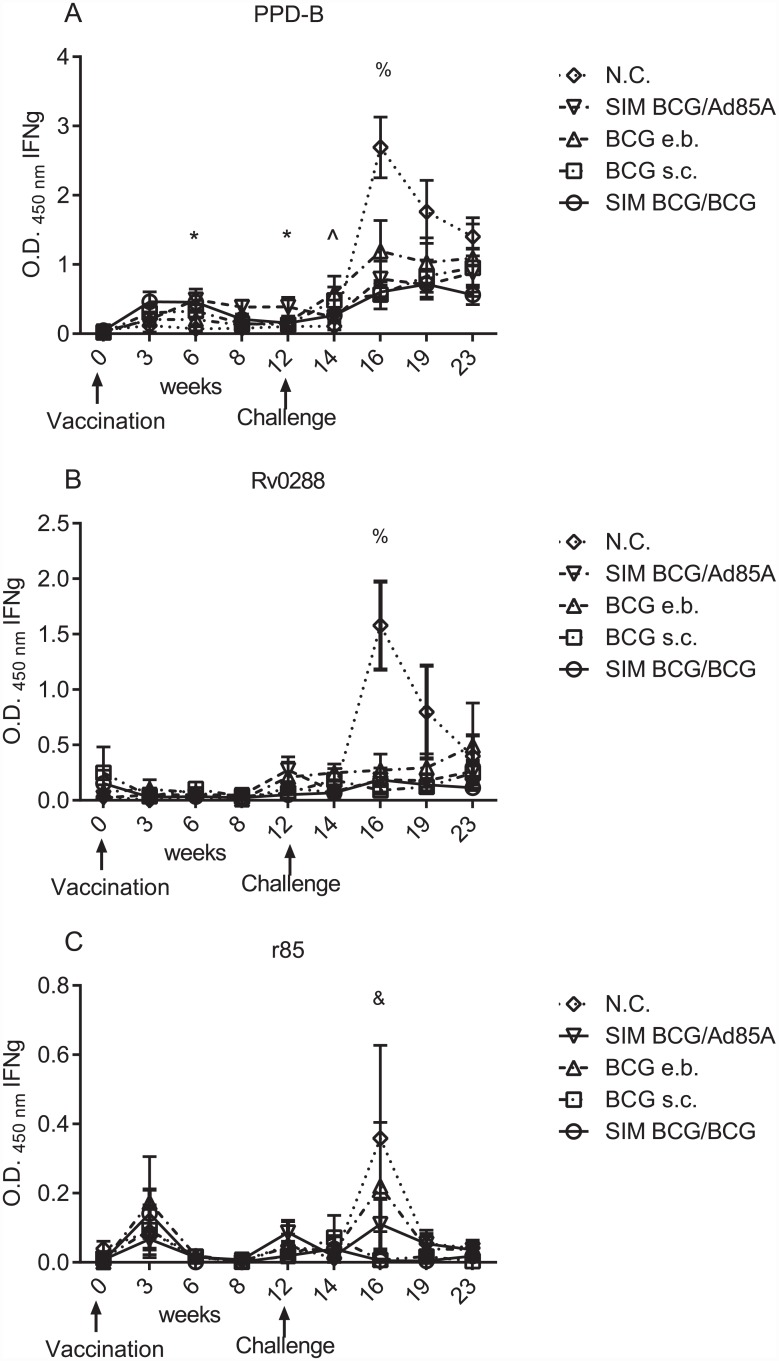
Different vaccination regimes induced different responses to mycobacterial antigens. Determination of the secretion of IFNγ by whole blood cells, measured by ELISA, from animals vaccinated with BCG at week 0 and challenged with *M*. *bovis* at week 12; vaccination and challenge are indicated by arrows on the *x* axis. Group average of antigen-specific whole blood IFNγ secretion, expressed as OD 450nm, was corrected for background and evaluated at the weeks indicated on the *x* axis. Animal groups are indicated naïve controls (N.C.) (open rhomboid); SIM BCG/Ad85A (inverted open triangle); BCG e.b. (open triangle); BCG s.c. (open square); SIM BCG/BCG (open circle). Error bars represent the standard error of the mean (SEM). Data were analysed using a using a Kruskal-Wallis test with Dunn’s multiple column post-test comparison. Statistical significance is indicated as follows: * difference between BCG/Ad85A and N.C.; ^ indicates differences between BCG e.b. and N.C.; % indicates differences between BCG s.c. and N.C.; & indicates differences between BCG/BCG and N.C. One symbol indicates ρ<0.05.

Fig 1 shows the antigen specific immune responses induced by different vaccination regimes, as determined by measurement of IFNγ with the Bovigam assay, following overnight culture of whole blood with mycobacterial antigens. Levels of PPD-B-induced IFNγ produced after BCG s.c., rose from week three onwards. At week 6 and 12, responses to PPD-B induced by BCG/Ad85A were higher than the responses observed in N.C. (ρ<0.05). In terms of kinetics, BCG s.c. induced responses to PPD-B at weeks 3 and 6 that were significantly different to those detected at week 0 (ρ<0.05); BCG/BCG and BCG/Ad85A also had responses to PPD-B at week 6 that were significantly different to those detected prior to vaccination at week 0 (ρ<0.05) (S1 Fig). Negligible levels of IFNγ were detected in naive control animals before challenge with bTB.

Twelve weeks after vaccination animals were infected with *M*. bovis. At week 14, only animals vaccinated with BCG e.b. showed a statistically different response to PPD-B compared to N.C. animals (ρ<0.05). In terms of kinetics, only animals vaccinated with BCG s.c. or BCG e.b showed responses to PPD-B at week 14 (ρ<0.05) and ρ<0.01, respectively). At week 16, the responses to PPD-B and Rv0288 detected in animals in the N.C. group were significantly different from the responses detected in animals vaccinated with BCG s.c. (ρ<0.05). Also at week 16, there were differences in the responses of animals in the N.C. group to r85 compared to responses in BCG/BCG vaccinated animals (ρ<0.05). Animals in all groups made responses to PPD-B from week 16 until the end of the experiment that were larger than those detected at week 0 (range of ρ<0.05, ρ<0.01, ρ<0.001). SIM BCG/Ad85 vaccinated showed responses to Rv0288 from week 16 until the end of the experiment that were larger than those detected at week 0 in the same animals (ρ<0.05 and ρ<0.01). At week 14, N.C. animal showed responses to Rv0288 that were larger than those observed at week 0 in the same group ρ<0.01).

**Fig 2:**
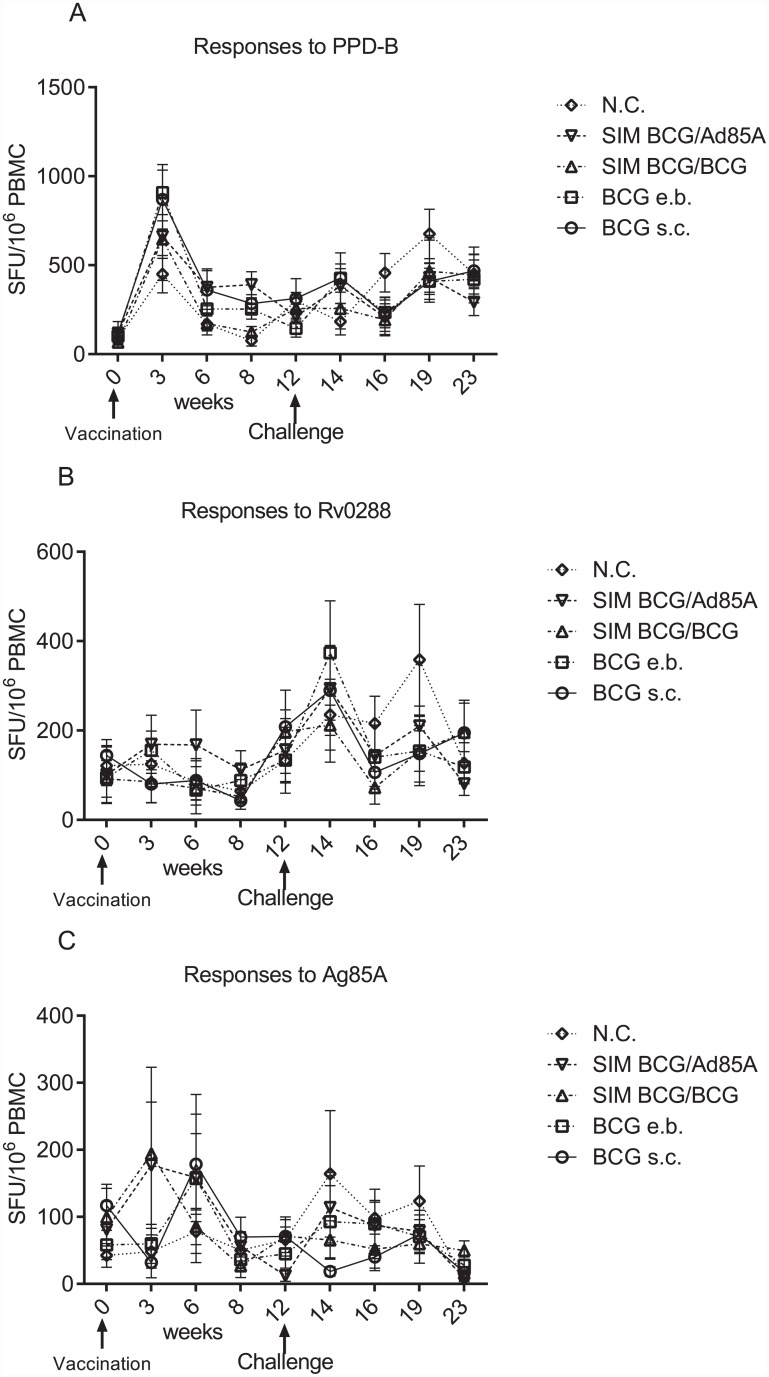
The comparative frequency of IFNγ secreting cells responding to mycobacterial antigens does not appear to be affected by different vaccination regimes. The frequency of IFNγ producers in PBMC, as determined by ELISpot, from animals immunised with BCG at week 0 and challenged with 2000 cfu *M*. *bovis* at week 12 was determined as indicated in materials and methods; immunisation and challenge are indicated by arrows on the *x* axis. IFNγ ELISpot was evaluated at the weeks indicated on the *x* axis, corrected for background and expressed as spot forming units (SFU) per 10^6^/PBMC. No significant differences in the frequency of IFNγ secreting cells responding to PPD-B, Rv0288 or r85A was detected between the different vaccination regimes after vaccination or after infection. N.C. (open rhomboid); SIM BCG/Ad85A inverted triangle; SIM BCG/BCG (open triangle); BCG e.b. (open square); BCG s.c. (open circle). Error bars represent the standard error of the mean (SEM). Data were analysed using a using a Kruskal-Wallis test with Dunn’s multiple column post-test comparison.

Fig 2 shows the frequency of IFNγ producing cells after vaccination and infection. None of the vaccination regimes induced responses to any of the antigens used greater than those observed in the non-vaccinated group, however animals vaccinated with BCG s.c. (ρ<0.001), SIM BCG/BCG (ρ<0.01) and SIM BCG/Ad85 (ρ<0.01) showed responses that were higher than those observed at week 0 (S2 Fig). After infection, as after vaccination, no differences were observed in the responses to mycobacteria antigens between the different treatment groups but BCG s.c. vaccinated animals showed responses to Rv0288 that were different to those observed at week 0 (ρ<0.05). At week 19, N.C. animals (ρ<0.05) and those vaccinated with BCG e.b. (ρ<0.01) or SIM BCG/Ad85 (ρ<0.05) showed responses to PPD-B that were different from those observed at week 0. Responses to PPD-B detectable at week 23 in BCG e.b. and SIM BCG/BCG vaccinated animals were different to those detected at week 0 (ρ<0.05).

### Pathological analysis

**Fig 3:**
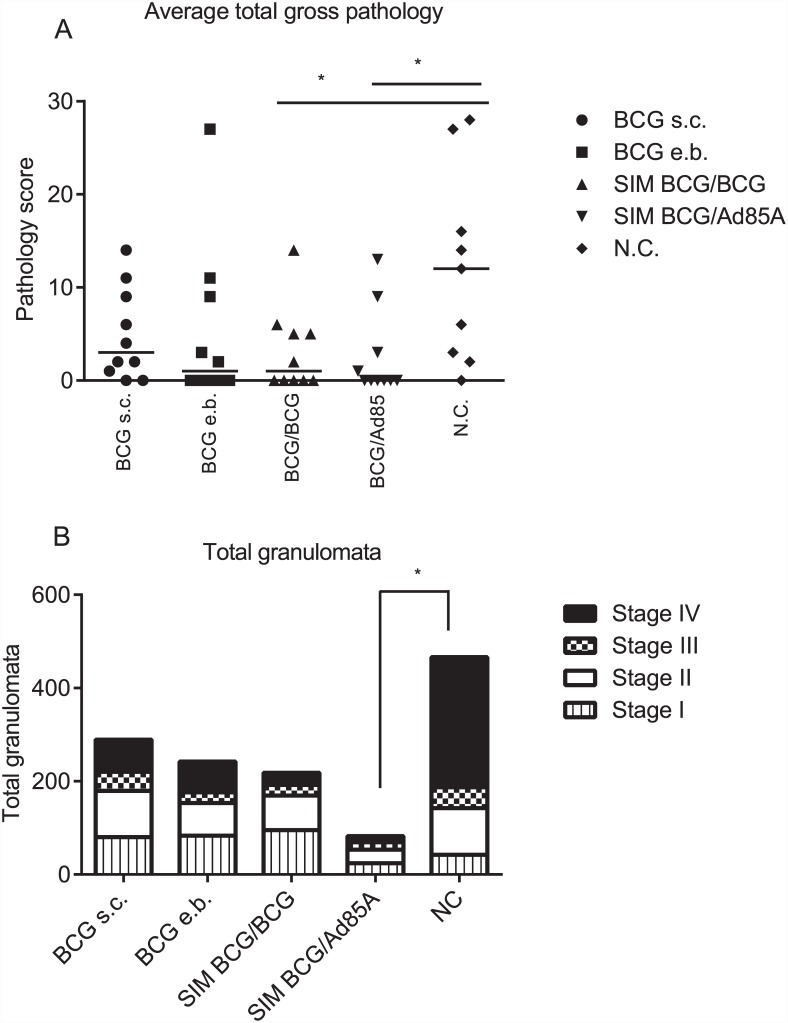
Evaluation of pathology for each group of treated cattle. Treatment groups are indicated on the *x* axis. Summary of total visible pathology scores for each group evaluated as previously described [20] **(A)**. Summary of the number of granulomata at different developmental stages for each experimental group in thoracic lymph node samples isolated at post-mortem from *M*. *bovis* infected animals. Granulomata were classified as previously described [21]; vertically hatched boxes; stage I granulomata, open boxes; stage II granulomata, dotted boxes; stage III granulomata, black boxes; stage IV granulomata **(B)**. Bar spanning groups BCG/BCG and N.C. indicates statistically significant difference between these two groups; Bar spanning groups BCG/Ad85A and N.C. indicates statistically significant difference between these two groups the indicated groups; * indicates ρ<0.05.

Fig 3 summarises the pathological findings in all five groups of animals 24 weeks after the initial vaccination, *i*.*e*. 12 weeks after infection. Fig 3A shows the total pathology scores of lungs and respiratory tract associated lymph nodes, for each experimental group. The group with the largest number of animals showing no visible lessions was SIM BCG/Ad85 with 6/10 animals showing no visible lessions; followed by animals in the SIM BCG/BCG and BCG e.b. groups with 5/10 in each. In BCG s.c. vaccinated animals 2/10 animals showed no visible lesions and in naïve controls only 1/10 infected animals showed no visible lesions. This assessment of visible pathology indicates that SIM BCG/BCG and SIM BCG/Ad85A animals had significantly lower pathology scores than naïve controls (ρ<0.05). There was a trend for all the vaccinated groups to have reduced pathology compared with naïve controls but the difference was not significant when comparing animals from BCG s.c. and BCG e.b. groups to naïve.

Fig 3B shows the outcome of histopathological evaluation of the different stages of granuloma formation in thoracic lymph nodes [21]. We consider granulomata type III and IV as advancing granulomata in which the host is unable to control the infection; comparison of the sum of type III and IV granulomata in the different groups showed that only SIM BCG/Ad85A animals had significantly lower number of these granulomata compared to naïve controls (ρ<0.05).

## Discussion

Intradermal Ad85A enhances protection against *M*. *bovis* infection in cattle when used in a heterologous prime-boost regime with subcutaneous BCG [11]. Similarly, Ad85A administered i.n. to guinea pigs or mice enhances the protective effect of BCG against pulmonary *Mtb*, in both species more effectively than intradermal or intramuscular boosting [7, 10]. In mice, immunisation directly into the lung primes local memory immune responses and is effective because local immune responses can inhibit the early phase of *Mtb* growth, while intradermal or subcutaneous immunisation inhibits *Mtb* growth only later after infection [14]. These effects are additive and not a result of prime-boosting of the immune system, since the systemic and pulmonary vaccines can be unrelated antigenically and can be administered simultaneously [13]. Both SIM BCG/BCG and SIM BCG/r85A, increased protection compared to parenteral BCG immunisation alone [13].

Here we show that vaccination of cattle with SIM BCG/BCG or SIM BCG/Ad85 confers a degree of significantly enhanced protection against challenge with *M*. *bovis*. Of interest, only animals in the SIM BCG/Ad85A showed statistically significant differences in the number of type III and IV granuloma compared to naïve controls and this group was also had the highest number of animals with no visible lesions after infection.

Whilst the SIM BCG/BCG and SIM BCG/Ad85A animals did not show a statistically significant difference from the BCG s.c. or e.b. immunised cattle, these two groups were the only ones to show a statistically significant difference from the naïve group. In terms of numbers of type III and IV granulomata, which we consider to indicate that the host is unable to contain the growth of mycobacteria, only the SIM BCG/Ad85A group showed a statistically significant difference from the naïve group. These observations are reminiscent of the improved protection against TB found in mice immunised simultaneously with BCG/r85A [13] and suggests that e.b. mucosal immunisation induces local responses that may contribute to the control of *M*. *bovis* by the host and a reduction in pathology, compared to the other immunisation regimes. It is of interest that the number of animals presenting no visible lesions did not match the number of animals harbouring mycobacteria (please see S1 Table) i.e. animals with no visible lesions or granulomas were positive for mycobacterial growth (due to the discreet nature and uneven distribution of lesions in bovine TB it is not possible to present biologically meaningful quantitative data; hence data are presented in a qualitative form).

The patterns of responses measured by the Bovigam^™^ and IFNγ ELISPOT varied between the different groups but it is not possible to state that one immunization regime induced stronger or weaker IFNγ responses to PPD-B than another. These data confirm that evaluation of IFNγ on its own is not a correlate of protection and highlights the need for the development of correlates of protection against tuberculosis.

In this occasion, the level of protection conferred by BCG s.c. compared to naïve controls was not significant and perhaps this is a reflection of the variability of protection conferred by BCG in an outbred species. Although not statistically significantly different, nevertheless, it is of interest that in a head to head comparison, BCG e.b. is at least as effective as BCG s.c. at reducing the severity of disease in terms of visible pathology and histopathology. Further studies are needed to examine whether pulmonary administration of BCG is more efficient in protecting against bTb infection and transmission. Furthermore, since in cattle parenteral prime boosting with Ad85A is effective [11], while in mice and guinea pigs pulmonary boosting is greatly superior [7, 10] it will be interesting to further investigate the mechanism(s) of protection afforded by local vaccination with Ad85A in cattle.

From a practical immunisation standpoint, it will be important to determine whether mucosal is more effective than parenteral boosting in cattle and whether simultaneous immunisation, which has the advantage that it can be performed at one time, is more effective than heterologous prime boosting approaches. Finally, although mice are protected for several months by Ad85A [14], the duration of protection induced by prime boosting or simultaneous immunisation with BCG s.c. and Ad85A given by the pulmonary route, remains to be determined. Clearly if SIM or parenteral/mucosal prime boost regimes are shown to be more effective than purely parenteral regimes, safe, practical and effective means for immunising the lungs will be required for field implementation.

## Supporting Information

S1 Fig Subcutaneous inoculation with BCG induces responses to PPD-B. Determination of the secretion of IFNγ by whole blood cells, measured by ELISA, from animals vaccinated with BCG at week 0 and challenged with *M*. *bovis* at week 12; vaccination and challenge are indicated by arrows on the *x* axis. Group average of antigen-specific whole blood IFNγ secretion, expressed as OD 450nm, was corrected for background and evaluated at the weeks indicated on the *x* axis. Responses to PPD-B (closed circles) Rv0288 (open triangles) and r85A (open diamonds) are indicated at the same time points. Each graph represents the responses to the different antigens of animals in each group: **A**: BCG s.c., **B**: BCG e.b., **C**: SIM BCG/BCG, **D**: SIM BCG/Ad85A and E: naïve controls. Error bars represent the standard error of the mean (SEM). Data were analysed using a Friedman test with Dunn’s multiple column post-test comparison Statistical significance is indicated by * for PPD-B; ^ for Rv0288. *** = ρ<0.001; ** or ^^, = ρ<0.01; * or ^ = ρ<0.05.
(EPS)

S2 Fig Subcutaneous vaccination with BCG induces PPD-B specific IFNγ secretors. The frequency of IFNγ secreting cells in response to PPD-B (closed circles) Rv0288 (open triangles) and r85A (open rhomboid) are shown at the indicated time points. Each graph represents the responses to the different antigens of animals in the different groups: BCG s.c., BCG e.b., SIM BCG/BCG, SIM BCG/Ad85A and naïve controls (A-E, respectively). Error bars represent the standard error of the mean (SEM). Data were analysed using a Friedman test with Dunn’s multiple column post-test comparison. Statistical significance is indicated by * for PPD-B; ^ for r85A; and + for Rv0288. ***, ^^^ or +++ = ρ<0.001; **, ^^, ++ = ρ<0.01; *, ^ or + = ρ<0.05.
(EPS)

S1 File GraphPad Prism 6 file containing readings for average O.D._450_ nm readings of Bovigam Elisa performed with supernatants of whole blood cultures that had been stimulated as indicated in materials and methods. (PZFX)

S2 File GraphPad Prism 6 file containing data derived from ELISPOT experiments. Elispot counts obtained after multiplication of original data by 5 to obtain spot forming units/10^6^ PBMC. Cultures were set up with 1x10^6^ PBMC/well in a final volume of 200 μl with stimulating antigen as indicated in materials and methods.
(PZFX)

S3 File GraphPad Prism 6 file containing total pathology scores for individual animals and granulomata counts per group as determined by histopathology. Pathology score and granulomata counts are presented in Fig 3A and 3B respectively.
(PZF)

S4 File Excel file containing pathology scores for individual animals divided into lung and lymph nodes. Total gross pathology counts for each animal in the different groups in the different tissues evaluated as indicated in materials and methods.
(XLSX)

S1 Table Bacterial load in the individual tissues of animals in the different treatment groups evaluated at postmortem as described in materials and methods. + indicates presence of *M*. *bovis*. - indicates no mycobacteria were recovered from these tissues.
(XLSX)
